# Next generation plasma proteome profiling of COVID-19 patients with mild to moderate symptoms

**DOI:** 10.1016/j.ebiom.2021.103723

**Published:** 2021-11-27

**Authors:** Wen Zhong, Ozlem Altay, Muhammad Arif, Fredrik Edfors, Levent Doganay, Adil Mardinoglu, Mathias Uhlen, Linn Fagerberg

**Affiliations:** a Science for Life Laboratory, KTH Royal Institute of Technology, Stockholm, Sweden; b Department of Clinical Microbiology, Dr Sami Ulus Training and Research Hospital, University of Health Sciences, Ankara, Turkey; c Department of Gastroenterology, Umraniye Training and Research Hospital, University of Health Sciences, Istanbul, Turkey; d Centre for Host-Microbiome Interactions, Faculty of Dentistry, Oral & Craniofacial Sciences, King's College London, London, United Kingdom

**Keywords:** COVID-19, Protein profiling, Plasma proteome, Immune response, COVID-19, Corona virus disease 2019

## Abstract

**Background:**

COVID-19 has caused millions of deaths globally, yet the cellular mechanisms underlying the various effects of the disease remain poorly understood. Recently, a new analytical platform for comprehensive analysis of plasma protein profiles using proximity extension assays combined with next generation sequencing has been developed, which allows for multiple proteins to be analyzed simultaneously without sacrifice on accuracy or sensitivity.

**Methods:**

We analyzed the plasma protein profiles of COVID-19 patients (n = 50) with mild and moderate symptoms by comparing the protein levels in newly diagnosed patients with the protein levels in the same individuals after 14 days.

**Findings:**

The study has identified more than 200 proteins that are significantly elevated during infection and many of these are related to cytokine response and other immune-related functions. In addition, several other proteins are shown to be elevated, including SCARB2, a host cell receptor protein involved in virus entry. A comparison with the plasma protein response in patients with severe symptoms shows a highly similar pattern, but with some interesting differences.

**Interpretation:**

The study presented here demonstrates the usefulness of “next generation plasma protein profiling” to identify molecular signatures of importance for disease progression and to allow monitoring of disease during recovery from the infection. The results will facilitate further studies to understand the molecular mechanism of the immune-related response of the SARS-CoV-2 virus.

**Funding:**

This work was financially supported by Knut and Alice Wallenberg Foundation.

**Research in context**

**Evidence before this study**

COVID-19 is a highly contagious disease. Many studies have suggested that cytokine storms and immunosuppression are highly associated with progression of the disease. It is therefore interesting to analyze the host response upon infection using plasma proteome profiling with a focus on the immune response related to the severity of the disease. Recently, several studies have been published in which blood protein profiles of patients with severe disease have been investigated using proteomics-based analysis and these studies have identified proteins elevated upon infection, including immune related proteins such as cytokines and interferons.

**Added value of this study**

Here, we have extended these earlier studies to include also patients with mild and moderate disease. A “next generation” plasma profiling strategy has been performed based on proximity extension assay followed by next generation sequencing targeting close to 1500 blood proteins. In addition, we sampled the same individual both when they were diagnosed with the disease as well as after 14 days, providing a comprehensive and longitudinal dataset of this less studied patient group as compared to the many studies focusing on severe and critical COVID-19 cohorts. More than 200 proteins were found to have significantly different plasma levels at the time of infection as compared to 14 days later. A comparison with the plasma protein response in patients with severe symptoms shows similar plasma protein profiles independent of symptoms, but with some interesting differences. The study demonstrated the usefulness of “next generation plasma protein profiling” to identify molecular signatures of importance for disease progression and to allow monitoring of disease during recovery from the infection. The results will facilitate further studies to understand the molecular mechanism of the host immune-related response of the SARS-CoV-2 virus and all data from our COVID-19 cohort are available for further studies.

**Implications of all the available evidence**

The study shows that older individuals have a slower recovery back to normal plasma levels after infection and the study demonstrates that many of these older patients display a “disease profile” even after 14 days of diagnosis, despite having no symptoms of disease. An interesting protein shown to be elevated in the infected patients is the host cell receptor protein SCARB2 involved in entry of other viruses, but not yet implicated in cell entry for SARS-CoV2.

Alt-text: Unlabelled box

## 1 Introduction

Corona virus disease 2019 (COVID-19) caused by the severe acute respiratory syndrome coronavirus-2 (SARS-CoV-2) is a highly contagious disease. Patients infected with COVID-19 suffers from a large variation of symptoms caused by the host immune response, including substantial respiratory problems, acute coronary syndromes and metabolic dysfunction [1], [2], [3], [4]. The mechanisms behind the disease and why some remain asymptotic carriers while other patients experience severe disease with fatal outcome are poorly understood [5], however, many recent studies have suggested that cytokine storms and immunosuppression are highly associated with progression of the disease [6], [7], [8], [9].

An important effort to understand the biology of the host-virus response is to move towards comprehensive proteome profiling of host proteins in blood in response to viral infections, not only to understand the basis for disease, but also to facilitate precision medicine efforts aimed at stratification and monitoring of patients before and during therapeutic interventions [10], [11], [12]. The objective is thus to probe the circulating plasma proteome of individuals with sensitive and specific assays that can allow massive sample throughput. However, progress has been hampered by the challenge to allow the quantification of thousands of proteins across more than a billion range in concentrations, starting with minute sample volumes [10].

We have previously reported the stable and unique plasma proteome profiles in healthy individuals [12,13], based on the Proximity Extension Assay (PEA) method [14]. Recently, we have also shown that this can be extended for simultaneous analysis of many more targets by the introduction of massive parallel sequencing, here referred to as PEA-NGS, without sacrifice on accuracy or sensitivity [10]. This new approach for “next generation plasma profiling” allows for simultaneous analysis of close to 1500 protein targets from small volumes of samples, and facilitates sensitive multiplex assays to be coupled with low cross-reactivity and minimal off-target events, as exemplified by the analysis of type 2 diabetes patients [10]. Recently, Patel et al [15] and Filbin et al [16] described using the PEA analytical platform the plasma protein profiling of COVID-19 patients with a main focus on individuals with severe symptoms. Several liquid chromatography-tandem mass spectrometry (LC-MS/MS)-based studies have also investigated the plasma proteome upon mild and severe COVID-19 infection [17], [18], [19], [20]. These two platforms address the proteome using two different approaches, where LC-MS/MS is a system-wide and unbiased method whereas the PEA technology is a highly sensitive and targeted method [21]. Importantly, the two technologies can measure different populations of proteins, and although the overlap is inherently small, they generally support each other and show a high correlation when the same target is quantified across different individuals [21].

Here, we have used the PEA-NGS analysis to investigate the plasma proteome profile of COVID-19 patients with mild to moderate symptoms to allow comprehensive comparisons of protein responses as a result of infection. The results support previously published studies which mainly have involved severe or critical patient groups and our study allows for a comparison between protein profiling patterns in patient groups with difference in the severity of the symptoms. Here, we include the largest number of target proteins studied so far for the mild to moderate patient group and these are profiled at the onset of disease as well as after 14 days of recovery.

## 2 Methods

### 2.1 Participants

A total of 50 patients were randomly selected from a clinical trial cohort of 93 patients over 18 years of age, who had a positive nasal swab PCR test for COVID-19 within the previous 24 h and were in stable condition not requiring hospitalization [22]. Chest tomography was done to rule out pneumonia. Patients who had a partial oxygen saturation below 93% and required hospitalization after diagnosis were excluded. Treatment started on the day of diagnosis. All patients were tested for COVID-19 using PCR on day 14 and received a negative result.

Participants for the randomized, open-label, placebo-controlled, phase-2 study for evaluating the efficacy and safety of combined metabolic activators in COVID-19 patients were from the general Turkish population and recruited at the Umraniye Training and Research Hospital, University of Health Sciences, Istanbul, Turkey, from September 2020 to January 2021. The 50 selected patients were all in the placebo group of the study and received 2.5 gram of sorbitol as placebo. Written informed consent was obtained from all participants before the initiation of any trial-related procedures. The safety of the participants and the risk–benefit analysis were overseen by an independent external data-monitoring committee. The trial was conducted in accordance with Good Clinical Practice guidelines and the principles of the Declaration of Helsinki.

### 2.2 Ethics

Each participant provided informed written consent prior to the study. The study was approved by the ethics committee of Istanbul Medipol University, Istanbul, Turkey, and retrospectively registered at https://clinicaltrials.gov/ with Clinical Trial ID: NCT04573153. Patient information (patient number, date of birth, initials) was entered into the web-based randomization system, and the randomization codes were entered into the electronic case report form.

### 2.3 The wellness profiling study

The Swedish SciLifeLab SCAPIS Wellness Profiling (S3WP) program is based on the Swedish CArdioPulmonary bioImage Study (SCAPIS), which is a prospective observational study with 30,154 individuals enrolled at ages between 50 and 64 years from a random sampling of the general Swedish population [23] From 2015 to 2018 [10,12,13]. In total, 101 healthy individuals were recruited and the program was ongoing from 2015 to 2018 [10,12,13]. Extensive phenotype characterization of the subjects was conducted before the study to establish the inclusion and exclusion criteria for the definition of ‘healthy’ subjects. The exclusion criteria in the S3WP program included: 1) previously received health care for myocardial infarction, stroke, peripheral artery disease or diabetes, 2) presence of any clinically significant disease which, in the opinion of the investigator, may interfere with the results or the subject´s ability to participate in the study, 3) any major surgical procedure or trauma within 4 weeks of the first study visit, or 4) medication for hypertension or hyperlipidemia. The study is approved by the Ethical Review Board of Göteborg, Sweden (registration number 407-15). All participants provided written informed consent. The study protocol conforms to the ethical guidelines of the 1975 Declaration of Helsinki. As described before, a total of 76 subjects were randomly selected from the wellness study to investigate the plasma levels of proteins using PEA-NGS (Olink Explore) technology [10].

### 2.4 Plasma collection and processing

Blood samples (3 mL) were collected in EDTA containing tubes (Becton, Dickinson and Company, NJ, USA) using standard venipuncture protocols. Plasma was recovered by centrifugation and aliquoted samples were stored at −80 °C until analysis except one thaw-freeze step for virus inactivation. At this step, samples (45 μL) were allocated onto 96-well plates and were treated with 1% Triton X-100 (5μL) at room temperature for 2 hrs. Samples were stored at −80 °C until analysis.

### 2.5 Plasma protein profiling

Plasma proteins were analyzed using a multiplex Proximity Extension Assay (PEA) technology with high throughput sequencing readout (Olink Explore) [12,14]. As described before, the full library consists of specific antibodies targeting 1,472 proteins, comprising 1463 unique proteins, as well as 48 controls. Each antibody is labelled separately with unique PEA oligonucleotide probes, two separate and complementary sequences. The conjugated antibodies are mixed into four separate 384-plex panels (372 proteins and 12 internal controls used for QC and normalization) focused on inflammation, oncology, cardiometabolic and neurology proteins, respectively. The analytical performance of each of the protein assays included in the panel is carefully validated based on specificity, sensitivity, dynamic range, precision, scalability, endogenous interference and detectability (http://www.olink.com). Briefly, samples were randomized (different samples from the same individual were present within the same plate) and 2.8 µl of plasma were incubated overnight with antibodies conjugated to PEA probes at +4°C. Following the immune reaction, a combined extension and pre-amplification mix were added to the incubated samples at room temperature for PCR amplification. The PCR amplicons were thereafter pooled before a second PCR amplification step was performed with additions of individual sample index sequences. After pooling of samples, bead purification and QC of the generated libraries were followed on a Bioanalyzer. Finally, the sequencing was carried out using Illumina's NovaSeq 6000 instrument using two S1 flow cells with 2 × 50 base read lengths. Counts of known barcode sequences were thereafter translated into normalized protein expression (NPX) units through a QC and normalization process. NPX is a relative protein quantification unit on a log2 scale and values are calculated from the number of matched counts on the NovaSeq run. Data generation of NPX consists of three main steps: normalization to the extension control (known standard), log2-transformation, and level adjustment using the plate control (plasma sample). Specifically engineered internal controls were added to each sample and are utilized to reduce intra-assay variability. These include one immuno-based control (incubation step) using a non-human assay, one extension control (extension step) composed of an antibody coupled to a unique DNA-pair always in proximity and, also, one amplification control (amplification step) based on a double stranded DNA amplicon. In addition, each sample plate includes sample controls used to estimate the precision (intra- and inter-CVs). Three negative controls (buffer only) are utilized to set background levels and calculate limit of detection (LOD), three plate controls (plasma pool) adjust levels between plates (thus improving inter-assay precision, allowing for optimal comparison of data derived from multiple runs), and finally two sample controls (reference plasma) are included to estimate precision. After quality control, a total of 1459 proteins were included in the analysis. To assess the intra-platform variation of the plasma proteome profiling, we calculated the pairwise Pearson correlation between four Olink panels (inflammation, oncology, cardiometabolic and neurology) for three control proteins (IL6, CXCL8 and TNF) (Supplemental Fig 1a). The average Pearson correlation is 0.94, indicating high consistency of the measurements of protein levels. A pairwise correlation of all protein levels across the eight technical control samples also showed high reproducibility with a median Pearson correlation of 0.81 (Supplemental Fig 1b and 1c).

### 2.6 Normalization of the plasma proteome profiling data

To allow for comparison of the two cohorts, the protein expression profiles from the wellness study were normalized to the current study using intensity normalization based on control samples (n = 20, wellness study; n = 8, COVID-19 study) (see details in http://www.olink.com). In brief, 1) for each study and assay, the study specific median value was calculated based on all control samples; 2) for each assay, an assay-specific normalization factor was estimated by calculating the median level of the pairwise differences for each of the control samples; 3) for each assay in the wellness study, the assay-specific normalization factor was added to the original NPX value, to normalize it to the current study.

### 2.7 Statistics

All data analysis and visualization was performed using R (v3.6.3) [24]. The complete dataset has been included in Table S4. Differential expression analysis was carried out using multi-factor analysis of variance (ANOVA) method with the built-in R function “anova()”. Sex, age and BMI were included in the analysis as covariates. False discovery rate (FDR) was calculated by using p.adjust() function in R, which uses Benjamini−Hochberg method. Proteins with FDR < 0.01 were considered as differentially expressed proteins. Uniform Manifold Approximation and Projection (UMAP) [25] was performed based on scaled NPX values using the R packages umap [26]. The hierarchical clustering result visualized in dendrograms was based on Pearson correlation and created by first calculating a correlation matrix of Pearson's ρ between all analyzed samples. The correlation was converted to a distance metric (1 – ρ) and was clustered using the Ward2 algorithm. Circular dendrogram and radar chart were generated using R packages circlize [27] and fsmb [28].

### 2.8 Role of the funding source

This work was financially supported by Knut and Alice Wallenberg Foundation. The funder had no role in study design, data collection, data analyses, interpretation, or writing of report. The corresponding author had full access to all data in the study and held the final responsibility for the decision to submit for publication.

## 3 Results

### 3.1 The study cohorts

**Fig. 1:**
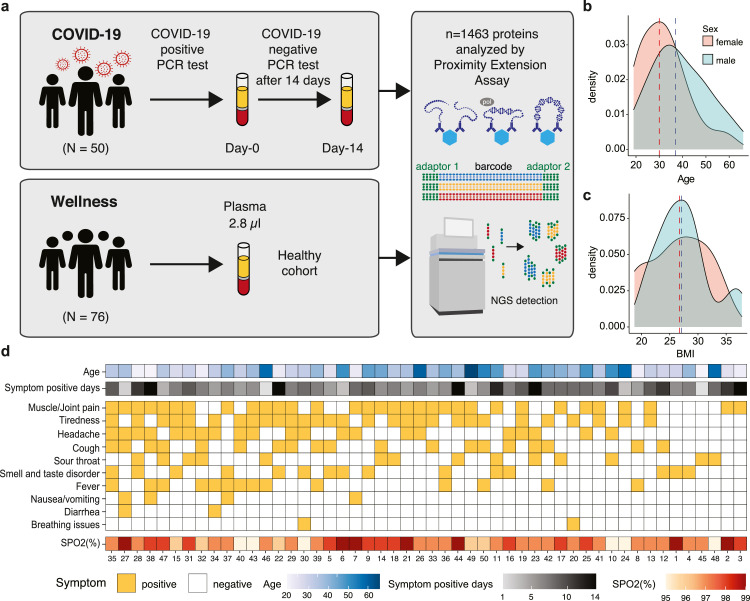
Overview of the study cohort. (a) The study design with the COVID-19 cohort as well as the wellness cohort from the S3WP program, which were both analyzed using the PEA-NGS method. (b) The age distribution of the COVID-19 cohort (n = 33, male; n = 17, female). (c) The BMI distribution of the COVID-19 cohort. (d) Heatmap showing the symptoms of each individual of the COVID-19 cohort as well as the age, symptom positive days and the measured oxygen saturation (SPO2) levels in percentage (%) (n = 50).

The analysis includes a cohort of 50 individuals with an ongoing COVID-19 infection and the plasma profiles in these individuals were compared with a healthy control population. Patients were recruited with a positive PCR test for SARS-CoV-2 and blood samples taken for analysis within 24 hours of confirmation of a COVID-19 infection (day-0) and exactly after 14 days (day-14). We have previously reported the individual plasma proteome variation in a healthy cohort with individuals between 50 - 65 years as part of the Swedish SCAPIS SciLifeLab Wellness profiling program (S3WP) [10,12,13], and this cohort was here used to allow for a comparison with a healthy control population. The study design is shown in Fig. 1a. The COVID-19 cohort consisted of individuals with a wide range in age (19 to 66) (Fig. 1b) with an average of 38 years and with an average body mass index (BMI) of 27 (18.8 to 37.8) (Fig. 1c). The number of days with symptoms after positive PCR-test is shown in Fig. 1d, with an average of 7.5 days. All individuals suffered from mild to moderate symptoms due to COVID-19 and a summary of the respective symptoms as well as the measured oxygen saturation (SPO2) levels for each person is visualized in the heatmap in Fig. 1d. A majority (78%) experienced muscle or joint pain or tiredness, whereas only 26% had fever and 4% had breathing issues (Table S1 and S2). None of them required hospital care, and at the second sampling time point on day 14, all had a negative PCR test.

### 3.2 Next generation plasma proteome profiling

**Fig. 2:**
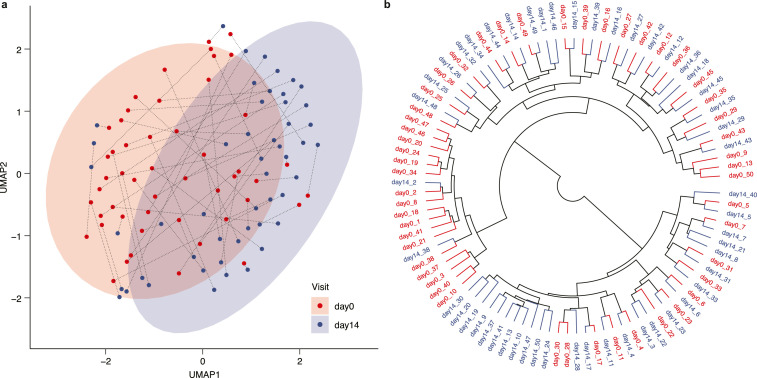
Clustering of COVID-19 samples. (a) UMAP plot showing the distribution of day-0 and day-14 samples, with each individual connected by a dotted line. (b) Dendrogram visualizing the results from hierarchical clustering of all samples. Day-0 samples are shown in red and day-14 samples in blue.

We used an approach for plasma protein profiling of the COVID-19 cohort where 1463 unique proteins were measured using the Olink platform (Olink® Explore 1536), which combines the PEA technology with Next Generation Sequencing (NGS) for read-out. The PEA-NGS technology allows for relative quantification of plasma protein expression levels which are calculated as Normalized Protein eXpression (NPX) values. A list with details about all analyzed plasma proteins that passed quality control (n=1459) is available in Table S3 and the complete table of NPX values for each protein is available in Table S4. In Fig. 2a, the expression profiles for each of the day-0 and day-14 samples based on all proteins were visualized using the dimensionality reduction method Uniform Manifold Approximation and Projection (UMAP) [25]. The resulting plot shows a separation between the two groups of samples, with most of the infection samples located together (red circle), but with some samples clustered at the individual level. The circular dendrogram in Fig. 2b shows the result from hierarchical clustering of samples colored by sample group. Here, we see two smaller clusters with mainly day-0 or day-14 samples clustered together, respectively, which indicates similar protein signatures within each group. However, in most cases each individual is most closely clustered with itself, supporting the previous reports stating that each individual has a unique and stable global proteome profile [10,13]. This is also evident in Fig. S2a, where the same dendrogram is colored by individual instead of sample group. A UMAP plot based on only day-0 samples shows that the global expression patterns cannot be explained by sex (Fig. S2b) or age (Fig. S2c) differences.

### 3.3 Analysis of the plasma protein response to infection

**Fig. 3:**
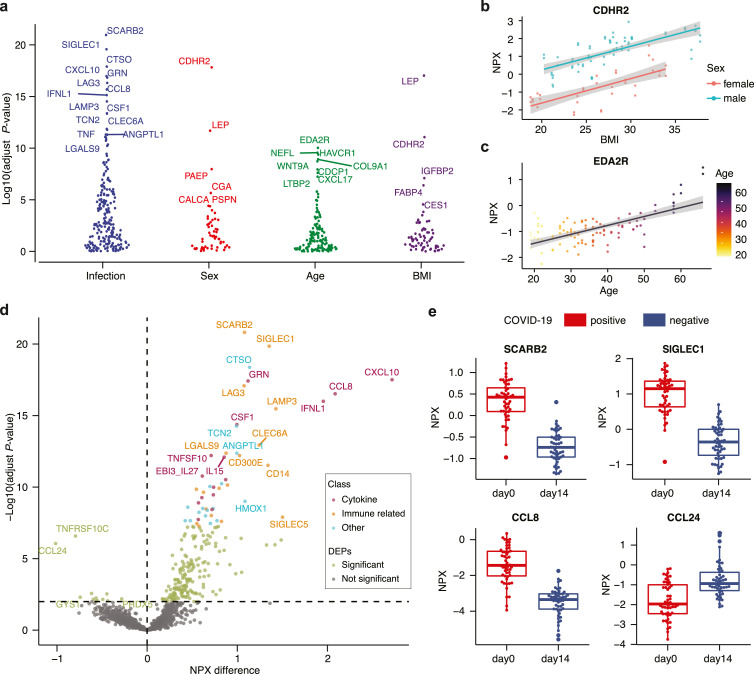
Proteins associated with COVID-19 infection. (a) Results from multifactor ANOVA based on the factors COVID-19 infection (day-0 / day-14), age, sex and BMI, showing the most highly associated proteins with each factor (n = 50). (b) Example of a BMI- (adjust *p* < 0.001) and sex-associated (adjust *p* < 0.001) protein cadherin related family member 2 (CDHR2) (n = 50). (c) Example of an age-associated protein ectodysplasin A2 receptor (EDA2R) (adjust *p* < 0.001, n = 50). (d) Volcano plot with differentially expressed proteins between day-0 and day-14 samples showing the difference in NPX values on the x-axis and -log10(adjusted *p*-value) on the y-axis (multifactor ANOVA with Benjamini Hochberg correction, n = 50). We show the manual annotation of the top 50 most significant proteins into groups of ‘cytokine’ (red), ‘immune related’ (orange) and ‘other’ (blue). (e) Boxplots of examples of up- and down- regulated proteins in day-0 samples (n = 50).

We performed a multifactor analysis of variance (ANOVA) for all 1459 proteins to discover the most highly associated proteins to COVID-19, while also taking into consideration the effects of age, sex and BMI (Fig. 3a and Table S5). The most significantly associated protein with COVID-19 disease is scavenger receptor class B member 2 (SCARB2), which is a host cell receptor protein involved in virus entry and has recently been described in the context of SARS-CoV-2 [15]. As expected from our previous studies [13], the most highly associated protein with BMI is leptin (LEP) (Fig. 3a). Cadherin related family member 2 (CDHR2) is the most significant sex-associated protein and is also associated with BMI (Fig. 3b). Ectodysplasin A2 receptor (EDA2R) (Fig. 3c) is most highly associated with age in our cohort supporting previous studies showing that this protein is linked to aging [29,30].

To further investigate which plasma proteins are most highly related to COVID-19 infection, we calculated the mean difference in expression and compared to the statistical significance based on the ANOVA results between the two groups of day-0 and day-14 samples for each protein. In the resulting volcano plot (Fig. 3d), all proteins with adjusted p-value < 0.01 are considered significant (n=239) and the full list is provided in Table S5. In addition, we performed a manual annotation of the biological function of the top 50 most significant proteins and classified them into three groups: (1) ‘cytokine’, (2) immune related’, or (3) 'other’ (Table 1). Interestingly, the scavenger receptor class B member 2 (SCARB2) (Fig. 3e), which is the most significant elevated plasma protein in the infected cohort, is reported as the cellular receptor for viral infection and responsible for viral entry [31]. Among the proteins differentially expressed in the COVID-19 infection samples almost all are up-regulated during the infection, including many proteins related to cytokine response, for example interferon lambda 1 (IFNL1) and the chemotactic factors C-X-C motif chemokine ligand 10 (CXCL10) and C-C motif chemokine ligand 8 (CCL8), which is known to play a role in neoplasia and inflammatory host responses (Fig. 3e). Proteins related to other immune-related functions were also found, including sialic acid binding Ig like lectin 1 (SIGLEC1), which functions as a macrophage-restricted adhesion molecule, and lymphocyte activating 3 (LAG3), which functions as an inhibitory receptor on antigen activated T-cells [32]. Only two proteins are found to be significantly down-regulated during infections: (i) the C-C motif chemokine ligand 24 (CCL24), which is a cytokine involved in the inflammatory response (Fig. 3e) and is a chemotactic for resting T-lymphocytes and eosinophils and (ii) the TNF receptor superfamily member 10c (TNFRSF10C), which is a receptor for the cytotoxic ligand TRAIL.

**Table 1:** Top 50 elevated plasma proteins in COVID-19 infected patients.

Protein	UniProt description	Classification	NPX difference	adjust *P*-value
SCARB2	scavenger receptor class B member 2	Immune related	1.08	1.5E-21
SIGLEC1	sialic acid binding Ig like lectin 1	Immune related	1.35	1.4E-20
CTSO	cathepsin O	Other	1.14	4.2E-19
CXCL10	C-X-C motif chemokine ligand 10	Cytokine	2.72	3.1E-18
GRN	granulin precursor	Cytokine	1.12	3.8E-18
LAG3	lymphocyte activating 3	Immune related	1.08	8.3E-18
CCL8	C-C motif chemokine ligand 8	Cytokine	2.09	3.0E-17
IFNL1	interferon lambda 1	Cytokine	1.95	1.0E-16
LAMP3	lysosomal associated membrane protein 3	Immune related	1.43	3.3E-16
CSF1	colony stimulating factor 1	Cytokine	1.00	4.3E-15
TCN2	transcobalamin 2	Other	0.99	5.4E-15
CLEC6A	C-type lectin domain containing 6A	Immune related	1.24	1.2E-13
ANGPTL1	angiopoietin like 1	Other	0.99	4.2E-13
LGALS9	galectin 9	Immune related	0.88	4.2E-13
CD300E	CD300e molecule	Immune related	1.03	6.2E-13
TNFSF10	TNF superfamily member 10	Cytokine	0.71	6.2E-13
IL15	interleukin 15	Cytokine	0.85	8.1E-13
CD14	CD14 molecule	Immune related	1.34	3.0E-12
EBI3_IL27	NA	Cytokine	0.61	1.7E-11
CX3CL1	C-X3-C motif chemokine ligand 1	Cytokine	0.87	3.0E-11
LGMN	legumain	Other	0.83	5.7E-11
CLEC4C	C-type lectin domain family 4 member C	Immune related	0.89	7.1E-11
TINAGL1	tubulointerstitial nephritis antigen like 1	Other	0.70	9.1E-11
CRLF1	cytokine receptor like factor 1	Cytokine	0.74	1.0E-10
PTX3	pentraxin 3	Immune related	0.80	1.2E-10
C1QA	complement C1q A chain	Immune related	0.55	1.4E-10
LILRA5	leukocyte immunoglobulin like receptor A5	Immune related	0.62	2.3E-10
IL18BP	interleukin 18 binding protein	Cytokine	0.73	3.5E-10
TNF	tumor necrosis factor	Cytokine	0.61	5.4E-10
HMOX1	heme oxygenase 1	Other	1.09	9.9E-10
IL18R1	interleukin 18 receptor 1	Cytokine	0.57	1.3E-09
ENTPD6	ectonucleoside triphosphate diphosphohydrolase 6 (putative)	Other	0.45	2.5E-09
VWA1	von Willebrand factor A domain containing 1	Other	0.62	3.1E-09
ESM1	endothelial cell specific molecule 1	Other	0.73	3.2E-09
DLL1	delta like canonical Notch ligand 1	Immune related	0.65	3.4E-09
TNFSF13B	TNF superfamily member 13b	Cytokine	0.72	3.6E-09
FOLR2	folate receptor beta	Other	0.67	4.2E-09
GAS6	growth arrest specific 6	Other	0.58	5.8E-09
LILRB4	leukocyte immunoglobulin like receptor B4	Immune related	0.71	9.6E-09
SEMA3F	semaphorin 3F	Other	0.65	1.0E-08
SIGLEC5	sialic acid binding Ig like lectin 5	Immune related	1.50	1.3E-08
TNFSF13	TNF superfamily member 13	Cytokine	0.57	1.8E-08
TPP1	tripeptidyl peptidase 1	Other	0.77	2.2E-08
ENTPD5	ectonucleoside triphosphate diphosphohydrolase 5	Other	0.42	2.2E-08
SMOC1	SPARC related modular calcium binding 1	Other	0.48	2.2E-08
BST2	bone marrow stromal cell antigen 2	Immune related	0.82	2.5E-08
FST	follistatin	Other	0.70	3.5E-08
VCAM1	vascular cell adhesion molecule 1	Immune related	0.55	3.6E-08
VSIG4	V-set and immunoglobulin domain containing 4	Immune related	0.71	4.1E-08
CD74	CD74 molecule	Immune related	0.58	5.4E-08

### 3.4 Comparing mild/moderate COVID-19 disease profiles with severe disease

**Fig. 4:**
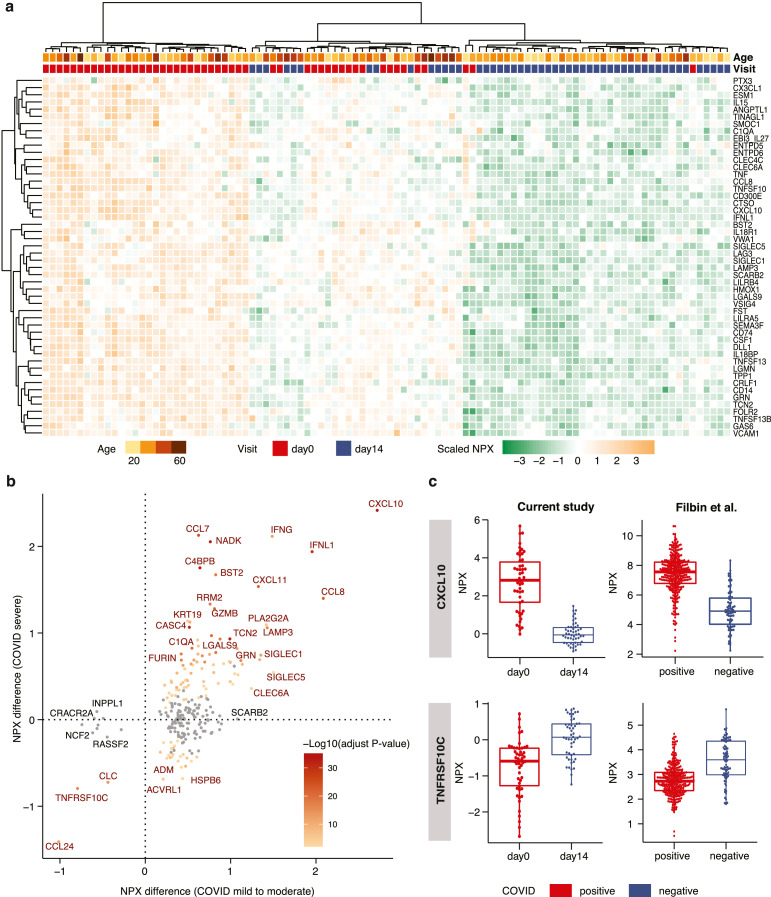
Analysis of the plasma proteins related to COVID-19 infection. (a) Heatmap showing the expression levels of the 50 most significant proteins in all day-0 and day-14 samples, clustered based on expression in the 50 proteins. (b) Scatterplot showing the difference in expression levels between day-0 and day-14 samples in our study on the x-axis, and the difference between COVID-19 positive and COVID-19 negative samples in the Filbin et al[16] study on the y-axis. All proteins with a significant difference in our study are shown. The color code depicts the -log10 adjusted *p*-value in the Filbin et al study, where the grey dots represent non-significant change. The statistical analysis is based on ANOVA with sex, age, bmi as covariates. (c) Boxplots of up- and down- regulated proteins in COVID positive and negative patients in both our study (n = 50) and in the Filbin et al study (n = 242, positive; n = 78, negative).

The expression levels of the 50 most significantly elevated proteins at COVID-19 infection (Table 1) are visualized as a heatmap in Fig. 4a. As expected, most of the samples at infection (day-0) have a similar plasma protein profile (left part of the heatmap), while most of the plasma profiles on day-14 cluster together (right). However, there are samples with intermediate plasma proteins elevated at COVID-19 infection (middle). These identified proteins elevated at infection (day-0) were compared with the proteins elevated in patients with severe symptoms (requiring hospitalization and oxygen supplementation, placed on mechanical ventilation or death) [16] (Table S6) and the comparison (Fig. 4b) shows that most proteins are elevated or down-regulated in a similar manner in patients with mild symptoms (this study) and severe symptoms [16]. An example of an elevated plasma protein at infection is the chemokine CXCL10 which is involved in the stimulation of monocytes, natural killer and T-cell migration (Fig. 4c). Similarly, an example (Fig. 4c) of a protein down-regulated in patients both with mild and severe symptoms is the TNF receptor (TNFRSF10C), which is a receptor for the cytotoxic ligand TRAIL involved in the cellular apoptosis. The comparison thus suggests a good correlation in host plasma protein response in patients with mild and severe symptoms. However, there are some notable differences, in particular the SCARB2 protein mentioned above, which do not show elevated levels in the cohort from the patients with severe symptoms[16]. There are also a group of proteins which are down-regulated in our study and do not show down-regulation in the patients with severe symptoms (Fig. 4b).

### 3.5 Comparison of the protein profiles between COVID-19 and healthy individuals

**Fig. 5:**
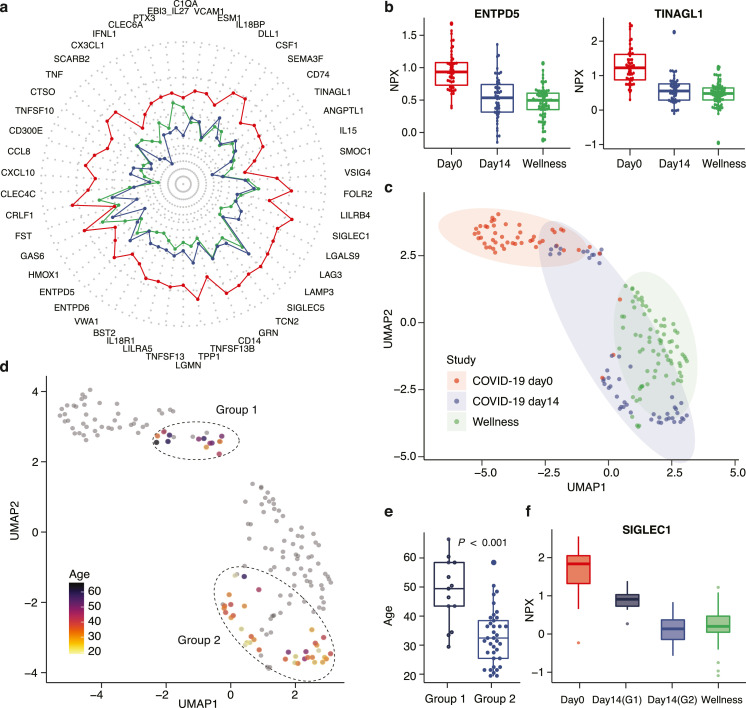
Analysis of the 50 most highly associated proteins to COVID-19 infection. (a) Radar plot showing the average expression levels of the 50 proteins for each of the three groups of samples (day-0 in red, color day-14 in blue color, and wellness healthy cohort in green color). (b) Boxplot showing the expression levels of two differentially expressed proteins ectonucleoside triphosphate diphosphohydrolase 5 (ENTPD5) and tubulointerstitial nephritis antigen like 1 (TINAGL1) in both the COVID-19 and wellness studies (n = 50, COVID-19 study; n = 76, wellness study). (c) UMAP plot showing the distribution of samples both from the COVID-19 cohort and the 76 samples from the wellness cohort based on the top 50 proteins. (d) UMAP plot highlighting the age of the individuals in the two different groups of day-14 samples. (e) Boxplot of the age distribution of the two different groups of day-14 samples and the p-value based on paired t-test (adjust *p* < 0.001, n = 50). (f) Boxplot showing the distribution of protein levels of a differentially expressed protein sialic acid binding Ig like lectin 1 (SIGLEC1) in day-0 samples, Group 1 (G1) and Group 2 (G2) of day-14 samples and the wellness cohort (n = 50, COVID-19 study; n = 76, wellness study).

Next, a comparison with a healthy cohort of individuals analyzed with the same analytical platform as part of a wellness study [10] was performed (Table S7). In Fig. 5a, the mean protein levels of the 50 most significant proteins (Table 1) in the infected patients (red) are shown as a radar plot and the levels are compared with the healthy individuals (green) and the same patients after recovery (blue). The results show the dramatic elevation of these proteins during acute infection, but also shows that in general these proteins have returned to healthy plasma levels after 14 days of diagnosis (recovery). Two examples of this include the proteins ectonucleoside triphosphate diphosphohydrolase 5 (ENTPD5) [33] and tubulointerstitial nephritis antigen like 1 (TINAGL1) [34], which have both been associated with COVID-19 severity (Fig. 5b). Next, a dimensionality reduction using UMAP was performed with the plasma profiles of the most significant proteins, including also the control group from the healthy population. The resulting UMAP plot (Fig. 5c) shows distinct clusters of samples from the infected patients (red) and the healthy control group (green). As expected, most of the samples from the patients after recovery (blue) shows a pattern similar to the healthy control group, but interestingly some of the individuals have protein profiles similar to the infected patients. In Fig. 5d, the same UMAP plot shows the individuals on day 14, color coded according to age. Interestingly, the majority of the individuals with an “infected plasma profile” after 14 days of diagnosis are older, suggesting a slower recovery in the older patients. In Fig. 5e, the age distribution of the first group (Group 1) with plasma profiles resembling infected individuals are compared with the second group (Group 2) with individuals who have plasma profiles resembling the healthy control group. This demonstrates that there is an age-related difference in response to the COVID-19 infection since many of the older patients, despite that they have no symptoms, are not fully recovered after 14 days based on this exploration of their plasma proteins. Furthermore, differentially expressed proteins between Group 1 and Group 2 were identified using ANOVA (Table S8). As an example, the sialic acid binding Ig like lectin 1 (SIGLEC1/CD168) protein, which is found on circulating monocytes in COVID-19 [35] and expression levels are associated with disease severity (Fig. 5f).

## 4 Discussion

Here, we present a comprehensive overview of the host response during a COVID-19 infection based on proximity extension assay combined with next generation sequencing read-out, providing a sensitive and accurate multiplex analysis of plasma proteins. We have analyzed close to 1500 human proteins in non-hospitalized individuals with mild to moderate disease. More than 200 proteins were found to have significantly different plasma levels at the time of infection as compared to 14 days later. An analysis of the 50 most significant plasma proteins (Table 1) demonstrates that a majority of the proteins with different plasma levels at COVID-19 infection are cytokine- or immune-related. Interestingly, the analysis shows that many of the older patients retain a plasma profile similar to the acutely infected patients still after 14 days of diagnosis, despite having no symptoms of disease. The results suggest that there is an age-related difference in plasma profile recovery in the older patients.

We have also compared our results with the analysis of patients with severe symptoms described recently [15,16]. The comparison shows that a majority of the proteins show similar response to the infection independent of the severity of symptoms, demonstrating no difference in host response despite dramatic differences in symptoms. Thus, many immune related proteins are elevated at infection in both cases, such as chemokine ligand 10 (CXCL10), interferon gamma (IFNG), interferon lambda 1 (IFNL1) and chemokine ligand 8 (CCL8). However, there are differences in response for some proteins depending on the severity of symptoms of the corresponding patients. Most notably is the protein scavenger receptor class B member 2 (SCARB2) which in our study is the most significant elevated protein at infection, but is not shown to be elevated in the severe patients according to Filbin et al [16], although the Patel study reported increasing levels of the protein in groups with severe (hospitalized and requiring oxygen supplementation) and critical (mechanical ventilation or death) COVID-19 disease [15]. SCARB2 is an interesting protein involved in membrane transportation and reorganization of endosomal and/or lysosomal compartments. This protein shows low tissue specificity (www.proteinatlas.org)[36] and studies have shown that the protein is involved in the pathogenesis of foot and mouth disease caused by enterovirus-71 and possibly by coxsackievirus A16. The question arises if this host cell receptor protein, involved in virus entry of enterovirus, could also be involved in SARS-CoV-2 entry into the cell. The role in COVID-19 is completely unexplored and these results suggest that more in-depth studies should be performed to explore its involvement in COVID-19 infections.

Of particular interest is also the sialic acid binding Ig like lectin 1 (SIGLEC1) protein, which is a macrophage-restricted adhesion molecule that mediates sialic-acid dependent binding to lymphocytes, and is a cell surface marker of interferon signaling. This protein is a member of the immunoglobulin superfamily and has previously been found to be upregulated by viral infection in macrophages [37]. Doehn et al [38] have recently reported that SIGLEC1is elevated in blood from patients with mild COVID-19 disease and that it is linked to the early phase of mild disease. Our data supports this observation and we also show that SIGLEC1 expression is associated with age and that the group 2 has much lower levels after 14 days of recovery.

Other interesting proteins found in our study are the cathepsin inhibitors, which have been shown to be associated with corona virus cell entry and replication [39]. These proteins are lysosomal peptidases involved in the endosomal pathway [39] and circulating levels of CTLS has previously been linked to disease severity of COVID-19 disease [40] and this protein has thus been proposed to be a promising therapeutic target [37]. In our study, we find that both the cathepsin L (CTSL) and cathepsin O (CTSO) proteins are elevated in plasma from patients with mild to moderate COVID-19 disease. Interestingly, the CTSL protein is also associated with age difference and they both show higher plasma levels in group 1 as compared to the group 2 both at diagnosis (day 0) and after recovery (day 14).

Here, we have used the highly sensitive and targeted proximity extension assay (PEA) for the analysis. Comparisons with earlier studies using mass spectrometry-based proteomics [17], [18], [19], [20] show overlap in many of the cytokine and immune-related proteins that have been identified here. This is reassuring since the two technology platforms target different concentration fractions of the proteome with rather limited overlap. The PEA provides a significant gain in coverage compared to the mass spectrometry-based assays, since it allows for profiling low abundant proteins down to pg/ml concentrations [21] despite low sample consumption [14]. The reproducibility and repeatability of PEA assays have also been investigated and found to perform well with high concordance between NGS and qPCR read-out [10]. However, a limitation with the PEA technology is the dependence on specific antibodies and thus only proteins with validated assays can be analyzed. Secondly, although we have carefully examined the intra-platform variation of the plasma proteome profiling using a combination of technical controls from both protein and sample level, it is important to point out that more external validation of these plasma protein profiles should be performed to promote their use as clinical biomarkers.

In conclusion, we here describe a comprehensive plasma protein analysis of COVID-19 patients with mild to moderate symptoms at the onset of disease as well as after 14 days, in the same individual. The analysis show that many proteins are elevated during COVID-19 infection and a comparison with earlier studies of patients with severe disease demonstrates similar plasma protein profiles independent of symptoms, but with some proteins differing in response. Interestingly, the analysis also reveals that older individuals have a slower recovery back to normal plasma levels after infection and the study demonstrates that many of these older patients display a “disease profile” even after 14 days after diagnosis, despite having no symptoms of disease. The study presented here demonstrates the usefulness of “next generation plasma protein profiling” to identify molecular signatures of importance for disease progression and to allow monitoring of disease during recovery from the infection. The results will facilitate further studies to understand the molecular mechanism of the host immune-related response of the SARS-CoV-2 virus.

## Contributors

MU, WZ and LF conceived and designed the analysis. AM, LD, MA, FE and OA collected and contributed data to the study. WZ, LF and MU performed the data analysis. MU, WZ and LF drafted the manuscript. All authors read and approved the final manuscript.

## Data sharing

The proteomic data of the COVID-19 cohort is available in the BioStudies database (http://www.ebi.ac.uk/biostudies) under accession number S-BSST719. The S3WP healthy cohort dataset has been deposited with the Swedish National Data Service (www.snd.gu.se, a data repository certified by Core Trust Seal): doi: 10.5878/rdys-mz27. This dataset can be made available for validation purposes by contacting snd@snd.gu.se. Data access will be evaluated according to Swedish legislation. Data access for research related questions in the S3WP program can be made available by contacting the corresponding author.

## Declaration of Competing Interest

The authors declare no competing interests.

## Appendix Supplementary materials

Image, application 1

Image, application 2

Image, application 3

Image, application 4

Image, application 5

Image, application 6

Image, application 7

Image, application 8

Image, application 9

Image, application 10
