# Supplementary material for: Next generation plasma proteome profiling of COVID-19 patients with mild to moderate symptoms
Source: eBioMedicine. 2021 Nov 27;74:103723. doi: 10.1016/j.ebiom.2021.103723 (PMC8626206; doi:10.1016/j.ebiom.2021.103723)
Supplement: Supplementary file 1 [file mmc1.docx]

**Supplementary Materials for**

Zhong et al. Next generation plasma proteome profiling of COVID-19 patients with mild to moderate symptoms

**Figures**

Fig. S1. Quality assessment of the proteome profiling

Fig. S2. Clustering of COVID-19 patients..

Fig. S3. Correlation between the 50 most significantly elevated proteins at COVID-19 infection

**Tables**

Table S1. Baseline demographics of the study population

Table S2. Description of the COVID-19 patients

Table S3. Full list of the analyzed plasma proteins

Table S4. Plasma proteome profiling of the COVID-19 patients

Table S5. Full list of the ANOVA results

Table S6. Comparison of the differentially expressed proteins in mild-to-moderate and severe COVID-19 patients

Table S7. Comparison between COVID-19 patients and healthy individuals

Table S8. Differentially expressed proteins between the two groups
