# Supplementary material for: Next generation plasma proteome profiling of COVID-19 patients with mild to moderate symptoms
Source: eBioMedicine. 2021 Nov 27;74:103723. doi: 10.1016/j.ebiom.2021.103723 (PMC8626206; doi:10.1016/j.ebiom.2021.103723)
Supplement: Supplementary file 2 [file mmc2.pdf]

Protein	UniProt description	Classification	NPX difference	adjust *P*-value
SCARB2	scavenger receptor class B member 2	Immune related	1.08	1.5E-21
SIGLEC1	sialic acid binding Ig like lectin 1	Immune related	1.35	1.4E-20
CTSO	cathepsin O	Other	1.14	4.2E-19
CXCL10	C-X-C motif chemokine ligand 10	Cytokine	2.72	3.1E-18
GRN	granulin precursor	Cytokine	1.12	3.8E-18
LAG3	lymphocyte activating 3	Immune related	1.08	8.3E-18
CCL8	C-C motif chemokine ligand 8	Cytokine	2.09	3.0E-17
IFNL1	interferon lambda 1	Cytokine	1.95	1.0E-16
LAMP3	lysosomal associated membrane protein 3	Immune related	1.43	3.3E-16
CSF1	colony stimulating factor 1	Cytokine	1.00	4.3E-15
TCN2	transcobalamin 2	Other	0.99	5.4E-15
CLEC6A	C-type lectin domain containing 6A	Immune related	1.24	1.2E-13
ANGPTL1	angiopoietin like 1	Other	0.99	4.2E-13
LGALS9	galectin 9	Immune related	0.88	4.2E-13
CD300E	CD300e molecule	Immune related	1.03	6.2E-13
TNFSF10	TNF superfamily member 10	Cytokine	0.71	6.2E-13
IL15	interleukin 15	Cytokine	0.85	8.1E-13
CD14	CD14 molecule	Immune related	1.34	3.0E-12
EBI3_IL2	NA	Cytokine	0.61	1.7E-11
CX3CL1	C-X3-C motif chemokine ligand 1	Cytokine	0.87	3.0E-11
LGMN	legumain	Other	0.83	5.7E-11
CLEC4C	C-type lectin domain family 4 member C	Immune related	0.89	7.1E-11
TINAGL1	tubulointerstitial nephritis antigen like 1	Other	0.70	9.1E-11
CRLF1	cytokine receptor like factor 1	Cytokine	0.74	1.0E-10
PTX3	pentraxin 3	Immune related	0.80	1.2E-10
C1QA	complement C1q A chain	Immune related	0.55	1.4E-10
LILRA5	leukocyte immunoglobulin like receptor A5	Immune related	0.62	2.3E-10
IL18BP	interleukin 18 binding protein	Cytokine	0.73	3.5E-10
TNF	tumor necrosis factor	Cytokine	0.61	5.4E-10
HMOX1	heme oxygenase 1	Other	1.09	9.9E-10
IL18R1	interleukin 18 receptor 1	Cytokine	0.57	1.3E-09
ENTPD6	ectonucleoside triphosphate diphosphohydrolase 6 (putative)	Other	0.45	2.5E-09
VWA1	von Willebrand factor A domain containing 1	Other	0.62	3.1E-09
ESM1	endothelial cell specific molecule 1	Other	0.73	3.2E-09
DLL1	delta like canonical Notch ligand 1	Immune related	0.65	3.4E-09
TNFSF13	TNF superfamily member 13b	Cytokine	0.72	3.6E-09
FOLR2	folate receptor beta	Other	0.67	4.2E-09
GAS6	growth arrest specific 6	Other	0.58	5.8E-09
LILRB4	leukocyte immunoglobulin like receptor B4	Immune related	0.71	9.6E-09
SEMA3F	semaphorin 3F	Other	0.65	1.0E-08
SIGLEC5	sialic acid binding Ig like lectin 5	Immune related	1.50	1.3E-08
TNFSF13	TNF superfamily member 13	Cytokine	0.57	1.8E-08
TPP1	tripeptidyl peptidase 1	Other	0.77	2.2E-08
ENTPD5	ectonucleoside triphosphate diphosphohydrolase 5	Other	0.42	2.2E-08
SMOC1	SPARC related modular calcium binding 1	Other	0.48	2.2E-08
BST2	bone marrow stromal cell antigen 2	Immune related	0.82	2.5E-08
FST	follistatin	Other	0.70	3.5E-08
VCAM1	vascular cell adhesion molecule 1	Immune related	0.55	3.6E-08
VSIG4	V-set and immunoglobulin domain containing 4	Immune related	0.71	4.1E-08
CD74	CD74 molecule	Immune related	0.58	5.4E-08
